# Oral Manifestations in Pediatric Patients with Coeliac Disease – A Review Article

**DOI:** 10.2174/1874210601711010539

**Published:** 2017-10-24

**Authors:** Viviana Marisa Pereira Macho, Ana Sofia Coelho, Diana Maria Veloso e Silva, David José Casimiro de Andrade

**Affiliations:** 1 Pediatric Dentistry, Faculty of Dentistry, University of Porto, Porto, Portugal; 2 Faculty of Medicine, University of Coimbra, Coimbra, Portugal; 3 Clinical Nutrition, Faculty of Nutrition and Food Sciences, University of Porto, Porto, Portugal; 4 Orthodontics and Pediatric Dentistry, Faculty of Dentistry, University, of Porto, Porto, Portugal

**Keywords:** Coeliac disease, Oral manifestations, Dental enamel defects, Recurrent aphthous stomatitis, Oral aphthous ulcers, Gluten

## Abstract

**Background:**

Coeliac disease is a chronic enteropathy that remains a challenge for the clinician, due to its atypical manifestations and etiopathogenic complexity.

**Objective:**

This article intends to describe the oral characteristics of Coeliac Disease in children in order to facilitate their management in the dental office.

**Methods:**

A review of the literature was performed electronically in PubMed (PubMed Central, and MEDLINE) for articles published in English from 2000 to April of 2017. The article is also based on the authors' clinical experience with children with coeliac disease. The searched keywords were “coeliac disease “,”oral manifestations “, “dental enamel defects”, “recurrent aphthous stomatitis” and “oral aphthous ulcers”.

**Results:**

There are some oral manifestations which are strictly related to coeliac disease: dental enamel defects, recurrent aphthous stomatitis, delayed tooth eruption, multiple caries, angular cheilitis, atrophic glossitis, dry mouth and burning tongue.

**Conclusion:**

The complete knowledge of the oral manifestations of coeliac disease can trigger an effective change in the quality of life of the patients with this disease.

## 1 INTRODUCTION

Coeliac disease (CD) is an immune-mediated enteropathy that affects genetically susceptible subjects following exposure to gluten in the diet [1-3]. Gluten is a proline-rich and glutamine-rich protein present in wheat (gliadin) barley (hordein) and rye (secalin) [4-6]. In different parts of the world the prevalence of CD has been estimated to be approximately 0.5%-1% [4, 7]. Current research in this area enables the detection of silent forms of the disease and makes it possible to assess its real prevalence, which is higher than initially thought [2].

This disorder is characterized by typical histological jejunal lesions showing total or subtotal villous atrophy, crypt hyperplasia and lymphocyte infiltration, which results in malabsorption of most nutrients in the small intestine [8, 9].

CD individuals may present extraintestinal or gastrointestinal symptoms (with a typical enteropathy, characterized by a malabsorption syndrome) or none of these [10, 11]. Atypical forms are also common and therefore often unsuspected [10, 12]. Due to the wide range of clinical manifestations oral lesions should not be overlooked [1, 12, 13].

## 2 METHODS

A review of the literature was performed electronically in PubMed (PubMed Central, and MEDLINE) for articles published in English from 2000 to April of 2017. The article is also based on the authors' clinical experience with children with coeliac disease. Extensive literature survey was conducted using the key words “coeliac disease”, “oral manifestations”, “dental enamel defects”, “recurrent aphthous stomatitis” and “oral aphthous ulcers”. A qualitative text analysis was done to select relevant scientific articles on the subject under study. The initial number of selected articles based on titles and abstracts was 54 and after a full text analysis 43 articles were included in the review.

## 3 RESULTS

A large prevalence of oral manifestations in patients with coeliac disease is described by numerous authors and some of the manifestations may be considered a diagnostic clue in silent-atypical forms of CD [14]. The oral manifestations strictly related to coeliac disease are: dental enamel defects, recurrent aphthous stomatitis, delayed tooth eruption, caries, geographic tongue, angular cheilitis, atrophic glossitis, burning tongue and dry mouth [14-16].

### 3.1 Oral Soft Tissue Lesions

#### 3.1.1 Recurrent Aphthous Stomatitis

Recurrent aphthous stomatitis (RAS) is the most common oral cavity inflammatory ulcerative condition, making its diagnosis and management a common problem in general and dental practice [17]. RAS is characterized by multiple recurrent small, round or ovoid ulcers with circumscribed margins, erythematous haloes and yellow or gray floors, typically presenting first in childhood or adolescence [17].

Though not fully understood, there are many factors associated to the etiology of RAS, including positive family history, local trauma, stress, immune changes, hormonal imbalance, food hypersensitivity and nutritional deficiencies [17-19]. This multifactorial disorder usually manifests itself in the non-keratinized oral mucosa and can cause considerable pain [20].

RAS is one of the most prevalent oral pathological conditions, affecting 10-20% of the general population and it’s more prevalent in children with nutritional deficiencies, immunodeficiencies, malabsorption and coeliac disease [17, 18, 21, 22].

Some authors suggest that RAS can be considered a sign of the atypical or the silent forms of CD. Campisi *et al.* completed a study with a group of 269 patients (3-17 years) with coeliac disease (confirmed both serologically and histologically) and compared them with a 575 healthy individuals group [10]. Aphthous-like ulcers were more frequently found in children with coeliac disease (22.7% *vs* 7.1% of controls) [10]. In the study of Procaccini *et al.* the prevalence of RAS was 26% in coeliac patients [23]. Bucci *et al.* found a prevalence of 33.3% of RAS in coeliac patients and reported that more than 1/3 of the coeliac subjects suffering from RAS benefited from a gluten-free diet [15].

In the study of Bramanti *et al*. RAS was found in 26/50 (52%) ascertained coeliac patients, 14/21 (66.7%) potential coeliac patients and 4/54 (7.4%) controls. This study also reported that RAS was more frequent in silent coeliac patients, who did not report any gastrointestinal symptoms before the diagnosis of CD [24].

This oral pathology usually occurs in the non-keratinized oral mucosa and affects the feeding, the speech and the swallowing as well as the tooth brushing. It also causes emotional instability because it can be the reason of considerable pain [20].

#### 3.1.2 Geographic Tongue

The geographic tongue is a chronic, inflammatory and immuno-mediated oral lesion with an unknown etiology [25]. Atrophy of the filiform papillae leaves an erythematous area with a white, yellow or grey elevated peripheral zone and irregular jagged pattern of the tongue [25]. This manifestation may occur secondarily to an iron, folic acid or vitamin B12 deficiency, resulting from intestinal malabsorption [26]. In the study of Bramanti *et al.* the geographic tongue was found in 5/50 (10%) ascertained coeliac patients, 4/21 (19%) potential coeliac patients, and 2/54 (3.7%) control subjects [24]. Cigic *et al.* found that 9 (15%) patients with geographic tongue were positive for IgA tTG and in those patients histological changes consistent with CD were confirmed by duodenal biopsy. Only two of the patients reported gastrointestinal symptoms [25].

#### 3.1.3 Atrophic Glossitis

Atrophic glossitis is an inflammatory disorder of the tongue mucosa. The tongue becomes smooth and with a shiny appearance with a red or pink background [27]. The smooth appearance is linked to the atrophy of filiform papillae that causes the development of circinate erythematous ulcer-like lesions on the dorsum and on the lateral border of the tongue [27]. The patients referred chewing, swallowing and/or speaking difficulties. This condition was noticed in the study of Bramanti *et al.* in 7/50 (14%) ascertained coeliac patients and 5/21 (23.8%) potential coeliac patients versus 1/54 (1.85%) controls [24].

#### 3.1.4 Angular Cheilitis

Angular cheilitis is a common condition characterized by erythema, ulceration, diffuse redness with an eroded area and crusting in the corners of the mouth [24]. This extra oral condition was recorded in the study of Bramanti *et al.* in 3/50 (6%) ascertained coeliac patients and 2/21 (9.5%) potential coeliac patients versus 2/54 (3.7%) patients of the control group [24].

#### 3.1.5 Burning Tongue

Burning tongue is characterized by oral tingling sensations, numbness and even burning sensation and severe pain, associated with objective signs of erythema and edema of papillae on the tip of the tongue not linked to any type of physical trauma [24]. It was registered in the study of Bramanti *et al.* in 7/50 (14%) ascertained coeliac patients, 2/21 (9.5%) potential coeliac patients and 3/54 (5.55%) control patients [24].

### 3.2 Oral Hard Tissue Lesions

#### 3.2.1 Dental Enamel Defects

Impairment of dental crowns mineralization may occur in numerous systemic diseases but the defects found in coeliac patients are highly specific [28-30]. Dental enamel defects are mainly characterized by pitting, grooving and sometimes complete loss of enamel [24, 31]. These specific enamel defects have to be symmetrically and chronologically detectable in all four sections of the dentition [30, 32]. Other enamel defects (discolorations, hypoplasia or opacities) that are not symmetrical nor chronological and that are not present in the same teeth of both hemiarches are considered unspecific. Dental enamel structural defects may be diverse and may contain hypoplasia (functional disturbances) as well as hypomineralization (qualitative disturbances) [33]. For the purpose of the assessment of those changes, Aine *et al.* (Table **1**) classified the specific enamel defects in grades I–IV according to the severity of their clinical aspect [24, 30-32].

The etiology of dental enamel defects in coeliac patients is not precisely clarified [34]. Some authors refer that the enamel defects could be related to hipocalcemia: the low serum levels of calcium resulting from intestinal malabsorption are decisive for the origin of enamel defects [35]. However, in a study by Wierink *et al.*, the number of enamel defects was higher in CD patients compared to the control group which also had intestinal malabsorption [35]. Another study mentioned that an autoimmune response against ameloblasts may be at the etiological origin of enamel defects in coeliac patients [35]. Others referred a particular genetic condition that leads to a specific immune response to gluten. Coeliac patients with HLA-DR3 genotype presented a higher risk of enamel lesions, pointing to a genetic etiology [35].

Malnutrition and vitamins D and A deficiency are also associated to enamel hypoplasia [36]. It is still unclear whether the oral lesions represent a direct manifestation of CD or whether they occur as a result of the indirect effects of malabsorption [36].

Enamel defects could be a major sign of CD [23, 29, 34]. The overall prevalence of this oral manifestation ranges from 9.52% to 95.94% [14, 29, 34]. Avşar and Kalayci reported that enamel defects were significantly higher in CD patients (42.2%) than in the control group (9.4%) [29, 34]. In the study of Wierink *et al.* enamel defects were found in 55% of the CD patients and in 18% of the control group [34, 35]. Campisi *et al.* observed specific dental enamel lesions in 60% of the patients with coeliac disease and only in 15% of the control group [10, 34]. A study of Cantekin *et al.* also reported a higher prevalence of enamel defects (48%) in CD patients compared to the control group (16%). The enamel defects in that study were generally symmetrical and mostly seen in anterior teeth [34]. In the study of Bramanti *et al.* the prevalence of specific enamel defects was 48% in ascertained coeliac and 19% in potential coeliac versus 0% in controls [24]. Costacurta *et al.* demonstrated that enamel hypoplasia was more prevalent in coeliac patients (33%) compared to healthy subjects (11%). The authors mentioned that the enamel defects found in coeliac individuals were more specific that the ones found in the control group; in fact many authors reported a symmetrical distribution, a chronologic coherence and the involvement of all dental hemi-arches [37]. A study by Bossu *et al.* reported that the enamel of CD patients was more fragile than the enamel of non-coeliac patients. The authors also described that the enamel prisms of the coeliac patients were short and highly hypomineralised, with an irregular distribution and with a smaller quantity of interprismatic substance when compared to that of non-coeliac patients [31].

The North American Society for Pediatric Gastroenterology, Hepatology, and Nutrition (NASPGHAN) included the presence of specific dental enamel defects as a risk factor for CD [4, 36].

#### 3.2.2 Dental Caries

Concerning dental caries there is some controversy.

Some studies reported that the prevalence of caries in CD was lower comparing to healthy subjects. A possible explanation for these results is the need for a carefully controlled diet. These patients maintain a rigid gluten free diet, which is a protein that can be found in several cariogenic foods, such as oatmeal, flours and breads, among others [30, 32, 38].

However, some authors reported a higher prevalence of caries in these patients. Hypoplasic enamel, changes in salivary composition and a low salivary flow rate are described and could constitute risk factors for dental caries [37]. The decrease of the salivary flow rate is related to the active phase of the disease and in concomitance with gluten-free diet. This decrease can cause dryness of the oral cavity and a burning tongue sensation, which can increase the risk for oral infections, including dental caries. Some authors have found that in a gluten-free diet regime coeliac patients had a low salivary concentration of amylase, IgA and IgM, a smaller volume of stimulated saliva, a lower buffering capacity and a lower calcium / phosphate ratio, which can influence the prevalence of dental caries [22, 38-40].

#### 3.2.3 Delayed Tooth Eruption

Prolonged malnutrition can have irreversible effects on tooth eruption. Since children with DC are often subject to weight loss and have lower somatic growth compared to healthy children, it is hypothesized that the development of the teeth is also subject to a delay [41].

Campisi *et al.* and Costacurta *et al.* observed a significantly more frequent occurrence of these disturbances in children with coeliac disease when compared to healthy children [10, 41]. According to Campisi *et al.*, delayed dental eruption may, similarly to the failure to thrive, be a signal of malnutrition and requires diagnostics toward gluten intolerance [10]. However, Mina *et al.* did not observe an association between the time of tooth eruption and coeliac disease [22].

The intra-oral observation of these patients presents as a cheap, quick and non-invasive procedure and the knowledge of coeliac disease’s oral manifestations may allow for the diagnosis of patients affected by silent-atypical forms of CD [37, 42]. Therefore, the oral manifestations can be the only presenting signs or symptoms in patients with undiagnosed CD [43]. When CD is suspected, dental practitioners should communicate with the physician so a screening for coeliac disease can be scheduled [37, 42, 43].

After diagnosis, all coeliac patients should be included in a preventive dental program aiming to provide professional oral hygiene, pits and fissures sealing, fluoride topical application and treatment of dental caries and fractures of hypoplasic enamel [37].

## CONCLUSION

Dental and oral manifestations such as dental enamel defects and recurrent aphthous ulcers are well-recognized manifestations of CD. Meticulous examination of the oral cavity with particular attention to these signs may contribute to an early diagnosis of coeliac disease.

The complete knowledge of the coeliac disease oral manifestations can trigger an effective change in the quality of life of these patients, propitiated by a joint and complementary work of a multidisciplinary team.

## Figures and Tables

**Table 1:** Classification of dental enamel defects in coeliac disease according to Aine [24, 30-32].

Grade 0	No Defects.
Grade I	Defect in enamel color. Single or multiple cream, yellow or brown opacities (marks) with clear or hazed boundary, part of the dental enamel may lack transparency.
Grade II	Slight structural enamel defects, rough surface with horizontal groves or pits, distortion of enamel color and transparency.
Grade III	Evident structural defects. A part or the entire surface of enamel rough and filled with deep horizontal grooves that vary in width or have large vertical pits; large opacities of different colors or strong discolorations may appear in combination.
Grade IV	Severe structural defects. The shape of the tooth changed. The tips of cusps are sharp-pointed and/or the incisal edges are unevenly thinned and rough. The thinning of the enamel material is easily detectable and the lesion may be strongly discolored.
